# How do roles impact suicidal agents’ obligations?

**DOI:** 10.1007/s11019-023-10177-5

**Published:** 2023-10-18

**Authors:** Suzanne E. Dowie

**Affiliations:** University of Glasgow, School of Law, Glasgow, G12 8QQ Scotland

**Keywords:** Suicide, Role responsibilities, Special obligations, Contracts

## Abstract

In this paper, I assess the role responsibility argument that claims suicidal agents have obligations to specific people not to kill themselves due to their roles. Since the plausibility of the role responsibility argument is clearest in the parent–child relationship, I assess parental obligations. I defend a view that says that normative roles, such as those of a parent, are contractual and voluntary. I then suggest that the normative parameters for some roles preclude permissible suicide because the role-related contract includes a promise to provide continuing care and emotional support. I propose that as we have established criteria for morally acceptable reasons for cancelling, voiding, or amending a contract, we can apply these to the role responsibility argument to establish grounds for releasing a parent from his role-related and contractual obligations. Failure to fulfil one’s contractual roles may not be blameworthy, depending upon the circumstances. I propose the factors determining culpability in failure to fulfil one’s role-related obligations are: intention, voluntariness, diminished responsibility, mental capacity, and foreseeability.

## Introduction

Even if we accept that suicide sometimes honours human intrinsic value, we must also consider that the impact of suicide on other people may be sufficient to render suicide morally impermissible; the reasons why suicide would otherwise be permissible may be abrogated due to someone’s relation to other people. It seems intuitively obvious that a parent who commits suicide violates her obligations to her dependent children to whom she is irreplaceable. This is the crux of the role responsibility argument that says suicide prevents us from fulfilling our role-related obligations to specific people; even if we do not have general duties to society not to kill ourselves, we nevertheless may have special obligations to particular people not to kill ourselves, by virtue of our role. This means that if a parent is suffering profoundly, her suicide may be at odds with her obligations to her children. The purpose of this paper is to clarify special obligations that suicidal agents may have to particular people in light of their roles. Since the plausibility of the role responsibility argument is clearest in the parent–child relationship, I assess parental obligations that preclude permissible suicide. To do this, in the section “The role responsibility argument,” I clarify the role responsibility argument. Then I go on to answer two related questions: (1) Who qualifies as being in the role of a parent? (2) What parental role-related obligations specifically preclude permissible suicide? As I shall show in this paper, this is a difference between someone who merely procreates (additionally, there are various ways of procreating, in part due to advances in medicine) and those who may not procreate at all but nevertheless qualify as a parent, as with adoptive parents. Once I have provided an account of who qualifies as a parent, I then go on subsequent sections to show what role-related obligations that arise from this account preclude permissible suicide.

To answer the first question, in the section “Parental roles,” I explain Michael Hardimon’s argument that many roles are contractual and voluntary. I then consider Elizabeth Brake’s proposal that parenthood is a voluntary undertaking, that mere biological procreators do not have the same obligations as those who voluntarily parent in the normative sense, but may owe compensation for negligently causing a child to exist. I disagree with Brake’s assessment that compensation owed should be equivalent to bringing it to a less needy condition until the age of thirteen, as opposed to normative parenting that requires more. I suggest that compensation may be equivalent to outsourcing those parental obligations that permissibly may be outsourced and should be proportionate with the degree to which one is culpable (for negligently causing a child to exist, for example). Even so, this leaves the problem of how to compensate a child for the loss of a parent who has committed suicide. Society, I argue, has a general duty of rescue to children who are harmed and/or wronged by their parents’ deaths, whether this death is a suicide or not, by providing a substitute normative parent.

To answer the second question, in the section “Parameters of the amended role responsibility argument,” I apply this view of normative parenting to the role responsibility argument. As role responsibilities are contractual, and as we have established moral bases for not fulfilling our contractual obligations under certain conditions, we likewise sometimes have a moral basis for not fulfilling our role-related responsibilities. I appeal to the conceptual framework of contracts to provide guidance with respect to the role responsibility argument. I propose that as we have established criteria, morally acceptable reasons, for cancelling, voiding, or amending a contract, we can apply these to establish grounds for not holding a parent to his role-related and contractual obligations. A parent who is terminally ill, for example, or has other morally compelling reasons for failing to fulfil her commitments, may permissibly kill herself, though she may nonetheless have obligations to her child; finding another suitable parent, for example. I then consider a possible objection to my proposal, that it does not allow adequately for nonvoluntarily acquired roles. While I do not deny the possibility that some roles are of this nature, I argue that the normative role of parenting is not of this type. One must accept the normative role of parent voluntarily, as opposed to the role of procreator that may be incurred contractually, nonvoluntarily, or negligently. I then make concluding remarks in the final section. Next, I explain the role responsibility argument.

## The role responsibility argument

Whilst we may have grounds for thinking that suicide is at least sometimes permissible or possibly even obligatory, we may nevertheless think that we have grounds for saying that suicide is sometimes impermissible. (Dowie 2020, 2022) (Note that throughout this paper, “*permissible* means that an act is not morally wrong. However, if an act of suicide is morally wrong, there is a sense in which it may still be *permitted by others,* if their intervention is not morally justified.” Dowie 2022; see also Dowie 2020.) Rather than claiming that we have a general obligation to society not to kill ourselves, the role responsibility argument says that suicide is impermissible because we have an obligation to provide specific goods or services to particular people based on our role in relation to those people. The specific goods that we provide depend upon the nature of the role that we have in relation to those particular people. Impermissibility, however, does not imply that we are justified in forcibly preventing suicide. (Dowie 2020, 2022).

Parenthood seems to be the paradigm function where the role responsibility argument is most obviously plausible. It seems intuitively clear that a healthy woman with dependent children wrongs those children when she kills herself without regard for their welfare. However, some other commonplace examples indicate that the role responsibility argument’s claim that suicide is always wrong may be false, even as it pertains to parents. For example, if a mother is diagnosed with a painful, terminal cancer and she makes adequate provisions for her children, then intuitively she is a permissible suicide. Alternatively, a parent suffering from locked-in syndrome may be a permissible suicide (if, for instance, assisted suicide is legalised in such cases, since she would not be able to kill herself on her own). For one thing, the parents in these cases are unable to care for their children anyway since they are very ill and this is beyond their control.

To accommodate these sorts of cases, we could weaken the role responsibility argument’s claim that suicide prevents us from fulfilling our role-dependent obligations to particular people, and doing so normally wrongs them. Accordingly, we end up with the following premises: (P1) If someone has role-related obligations, then it is normally impermissible for her to do things that undermine her obligations. (P2) Suicide undermines our ability to fulfil our role-related obligations. Conclusion: If someone has role-related obligations, then normally suicide is impermissible.

There are two senses of what it means to be in a role; one is descriptive and the other prescriptive or normative: (1) The descriptive sense of having a role is where the agent fulfils his role de facto; for example, people can be parents in this sense simply by having offspring. If roles are regarded this way, the role responsibility argument could make suicides impermissible even if a biological parent, for instance, is completely estranged from his children. (2) In the normative sense of having a role, the agent fulfils the role only if he performs it within certain normative parameters. One does not need to be a biological procreator to parent in this sense; one may take on a parenting role by administering to a child in certain ways, such as educating and socialising him, seeing to his physical welfare, and providing love and emotional support.

I maintain that a more adequate version of the role responsibility argument is going to be one that relies upon the prescriptive, normative conception of roles. If we think of roles in the normative sense, then the criteria of what it means to be a parent could be narrowed to include only those tasks and obligations that all parents who raise children ought to fulfil, for instance. Identifying these obligations could clarify the role responsibility argument’s parameters for suicide’s moral impermissibility and the conditions that constitute a normal case where a parent is obligated not to kill himself, as opposed to an exceptional case, where suicide may be morally permissible. To establish these parameters, I next clarify who occupies the normative role of parent and under what conditions.

## Parental roles

Since the role of parenting in the normative sense is arguably the paradigmatic role that most intuitively supports the role responsibility argument’s claim that suicide is impermissible, I now consider who qualifies to be a normative parental role-bearer. For instance, when is a biological procreator also a normative parent, and when is he not? In this section, specifically, I explain: (i) Michael Hardimon’s analysis that says that many roles, such as parenting, are contractual. (ii) I then consider Elizabeth Brake’s view, that merely biological procreators do not have the same obligations to children as ‘normative parents’ who acquire their special obligations voluntarily and contractually. (iii) I also consider Bernard Prusak’s objection that Brake should not regard procreators’ obligations as compensatory because, he says, restoring a child to its prior less needy state is nonsensical. He also thinks that the Frankenstein analogy shows that biological procreators have a duty to ensure a minimally decent life for their offspring, but part of this is providing love. (iv) I argue that compensation need not require restoring someone to his prior condition, but involves a substitution for a loss, and I show that Frankenstein’s failure was not that he failed to provide love, but was obligated not to procreate at all in the manner he did. I argue that Brake’s position is plausible, despite Prusak’s objections, but should be amended. (v) I compare and contrast the obligations of normative parents with other roles to determine what obligations preclude permissible suicide. I argue that contractually and voluntarily taking on continuing care and emotional support of someone is what makes suicide impermissible when it would otherwise be permissible.

### Hardimon’s view of roles as contractual

Michael Hardimon distinguishes roles as “constellations of institutionally specified rights and duties organized around an institutionally specified social function. Purely biological relations, considered merely as such, are not roles in my sense of the term.” (Hardimon 1994, p. 334) Hardimon explains that the standard view of roles is that they are either contractual or non-contractual, that contractual role obligations are “acquired by signing on for the roles from which they derive.” (Hardimon 1994, p. 337) These are entered into voluntarily and consist of an exchange of promises. (The standard basic form of contracts is: offer, acceptance, and consideration, or an exchange of promises that is voluntarily entered.) Both sides agree (promise) to the terms and each benefits from them. (Markovits 2015)).

In the voluntary and contractual sense of roles, Hardimon stipulates, in agreement with John Rawls, that “people have an obligation to carry out the tasks associated with their role providing (i) that the institution of which the role is part is just and (ii) that they have either voluntarily accepted the benefits of the institution or made use of the opportunities the institution provides to advance their own interests.” (Hardimon 1994, p. 335) In other words, these sort of roles are an exchange between social institutions and individual people within those institutions—people benefit from accepting the established role, and the institution (society) benefits from someone filling that role and providing the functions associated with it. In the role of parenting, for example, this would indicate that parents have contracts with society. (Children can be seen as third-party beneficiaries in parental contracts since they cannot voluntarily enter into such contracts.) Hardimon stipulates that such role-related obligations must be reasonable. (Hardimon 1994, p. 348) This grounds role-related obligations on justice and morality, rather than simply on social norms; otherwise, we may be obligated to perform tasks that are inherently wrong.

Hardimon says, “In signing on for a role, we promise to carry out the duties of the role, the tasks that the role requires.” (Hardimon 1994, p. 354) He suggests, however, that even if we do not wish to regard the terms of such contracts as promises, we may regard them as agreements to carry out specified tasks. (Hardimon 1994, p. 356) These roles are acquired voluntarily, either explicitly or tacitly. (Hardimon 1994, p. 357) Taking on roles, he notes, may happen gradually, as one may take on more and more role-related tasks. (Hardimon 1994, p. 357) A normative parent may accept the role tacitly, for example, when her partner expresses a desire to have children and she acts accordingly in order to bring that about; it is understood that she tacitly agrees though she does not necessarily verbally express her voluntary agreement. This means that if someone accidentally gets pregnant but does not have an abortion and decides not to give the child up for adoption but rather to raise her baby herself, she nevertheless has chosen to accept the normative role of parent.

One may object that this is not voluntarily accepting a role. Indeed, one may object that very few people are sufficiently informed about the nature of parenthood, and this seems to undermine the notion that parenthood is consensual, and therefore voluntary. This objection, however, is based on a particular conception of voluntariness that is seen, for instance, in medical consent. There are different conceptions and degrees of what it means to be acting voluntarily; the theoretical form of contracts does not require voluntariness in the sense of being ‘fully informed,’ such as with medical consent, that has developed a particular code for what it means to having consented voluntarily. Rather, in contract theory, the sense of voluntary acceptance may be more akin to an act of volition or free will. In terms of contracts, one may not be fully informed when purchases a shirt, for example, but one has nevertheless entered a contract. Likewise, when one enters a marriage contract, one may not know exactly what one is undertaking, but has nevertheless performed an act of volition necessary for a valid marriage (there may be exceptions to this, such as when someone has been duped into a marriage through fraudulent misrepresentation but depending on the circumstances such marriages cannot be said to be voluntary or valid). For more on voluntary action and its relation to knowledge, see Shand 1895 and Olsaretti 2004.)

Next, I assess Elizabeth Brake’s argument that normative parents have different obligations from biological procreators because they acquire such obligations voluntarily.

### Brake’s view of parental roles

Elizabeth Brake argues that there is a difference morally between procreating and fulfilling the social role of a parent, and the latter is only done voluntarily and within the socially constructed tasks related to parenting. She thinks that biological procreators do not have the same obligations as people who voluntarily parent in the normative sense. For example, a man who negligently or nonvoluntarily causes a child to exist may be obligated to ensure that she receives suitable parenting, but if he is unable or unwilling to be her normative parent, then he is not necessarily obligated to carry out the parenting-related tasks himself.

Brake argues that parental special obligations potentially could arise either voluntarily or as compensation for a wrong-doing. For instance, it is not necessary to be a biological procreator to be a normative parent; one may acquire special obligations when one voluntarily adopts a child. Alternatively, one may be a biological procreator because one is the proximate cause of a child’s existence through a wrong-doing, such as if one is negligent, and one may owe compensation. Brake acknowledges that we may have nonvoluntarily-acquired special obligations, such as those that children owe parents, as Hardimon suggests. She writes, however, that these may be explained by something else, such as virtues, and are “not correlative to moral rights.” (Brake 2010, p. 6) She claims that society establishes child-care responsibilities and normative parameters in such a way that they are special obligations that each parent has to his or her own children that are not owed to all children generally.

She argues that parenthood in the normative sense requires that one voluntarily take on the social and legal role, and this is conceptually different from causing a child to exist. She stipulates that voluntary acceptance of parental obligations is a necessary, but not sufficient condition of parenting in the normative sense because “at least two other conditions must obtain: that those taking on the obligations be able to carry them out, and that the child be eligible to be parented by them.” (Brake 2010, p. 3) This means that someone who is not capable of parenting should not do it (and would not perform within established parameters) and that people who steal others’ children, for example, are exempt from the moral, normative sense of parenting—they are not eligible to parent on social/legal terms.

In terms of compensation, she writes that there is “an explanatory gap between compensatory obligations entailed by moral responsibility for a child’s existence, or ‘procreative costs’ and moral parental obligations.” (Brake 2010, p. 8) Even if one has caused a harm (or wrong) by causing a child to exist, one must determine if, and how much, compensation is owed. This seems plausible if we consider that one may be a procreator who has proximate causal responsibility for bringing about a child’s existence, but would not owe any compensation; a commissioned carrying mother, a gamete donor, and a fertility clinician or obstetrician may be examples of this. Brake argues that the obligations to a child may amount only to a general duty of rescue, not to special parental obligations. She proposes that one incurs a special obligation of compensation if one has caused its being born in a needy condition and fails to provide it with a minimally decent life. (Brake 2010, p. 8) For example, if someone negligently fails to use adequate birth control methods and leaves the resulting child without adequate means of support, then she owes compensation due to her failure.

Brake claims that compensation normally involves the victim being brought back to his state before the harm had been done. This is not possible when we bring a child into existence, so compensation would be to “bring it to a less needy condition,” (Brake 2010, p. 9) or to compensate for having caused it to exist in a needy condition. Therefore, she thinks that if one is the biological procreator who causes a child to exist, all one has is an obligation to bring it to a less needy condition; to make sure that the child is not harmed (or wronged) until it is ‘less needy.’ She thinks that this means that compensation “could be discharged by providing food, shelter, and basic healthcare, and turning children out of doors as soon as they can survive independently—perhaps as young as 13.” (Brake 2010, p. 9).

Brake claims that compensation amounts to procreative costs and are not equivalent to parental obligations; this differentiates voluntarily acquired special obligations to parent from failure to fulfil a general duty of rescue (or not to harm others or allow them to be harmed) by abandoning a child. She argues that normative parental obligations are more than mere compensation that one may owe in the form of a certain amount of child support, for example; the role of a parent requires ensuring a decent life for a child past the age of independence, and parents also owe warmth and affection. Therefore, she claims that there is a ‘procreative gap’ between what nonvoluntary, negligent procreators and normative parents owe children. She maintains that evidence of this ‘procreative gap’ is that bringing a child to self-sufficiency and what constitutes a minimally decent life will vary with cultural norms. Parental obligations have increased in our culture, making them far more weighty than in others; parents bring children past a point of independence (Brake 2010, p. 9).

Since, Brake claims, normative parental roles do not generate compensatory obligations, she concludes that they must be voluntarily acquired. Brake says that if someone voluntarily takes on the role of ‘normative parent,’ more is required than mere procreative costs that involve a general duty of rescue. This includes such things as love and stability beyond a minimally independent state. Since parental obligations to children are voluntary, they do not require biological procreators to be involved in raising a child, and that many people may be involved in his rearing—the job could be community-based, for example. Brake thinks that this position makes sense of a parenting model where a village raises a child, for example.

Brake also argues that her view of parenting does not justify people just abandoning their children when we consider that if someone has voluntarily taken up the position, this is not equivalent to someone who has procreated by accident. She writes that the role of parenthood, once accepted, “cannot be contracted out with no moral cost, as compensatory obligations can. It matters, morally, that the role-holder perform role obligations herself.” (Brake 2010, p. 15) She says that once moral parents have begun to provide care, a child’s need for continuity requires exclusivity and “an agent cannot take on parental obligations which she cannot fulfil… this is a condition for occupying the role in the first place.” (Brake 2010, p. 18) Her view implies that a nonvoluntary biological procreator may permissibly commit suicide if he fulfils his obligation of compensation (for example, leaving money for the voluntary normative parents to raise the child) but a person who voluntarily parents in the normative sense could not permissibly commit suicide.

### Prusak’s objections to Brake’s position

Bernard Prusak claims that restoring a child to its prior less needy state is nonsensical. He writes, “the conclusion that should be drawn, I think, is that it does not then make sense to conceive of so-called procreative costs as ‘compensatory’—but Brake does not draw this conclusion, instead persisting in speaking of the child as being in a ‘harmed state’ for which procreators bear causal and moral responsibility.” (Prusak 2011, p. 64) Prusak also disagrees with Brake’s claim that there is a ‘procreative gap.’ He considers that standards have improved for children over the years, and that they can expect more from their parents now than they could two hundred years ago. While he agrees that parental obligations have increased, so too could it be said that procreative costs have also increased, and it is not clear that there is, in fact, a gap between the two.

He argues that since it is not safe to assume that there is a gap between procreative costs and parental obligations, procreation and parenting may entail the same obligations. He says that life can be a burden, even a curse, and a procreator has obligations beyond what Brake calls procreative costs of preventing harm. Procreators, he thinks, have an obligation to ensure that the lives of their progeny do not inspire the judgement that it would have been a mercy had they not been born. (Prusak 2011, p. 69) As such, it may be part of a procreator’s obligation of providing a minimally decent life to bestow normative role-related goods, such as unconditional love, as would be the case for a normative parent who voluntarily takes on the normative role. He thinks that the story of Frankenstein exemplifies this. (Prusak 2011, p. 69).

### My reply to Brake and Prusak

I propose that Brake’s view concerning compensation for some wrong-doing done to the child more clearly makes sense if we consider the following examples: (1) a fertility doctor negligently fails to screen an embryo, and as a result he proximately causes a child with severe abnormalities to exist. He would owe compensation to the normative parents to defray the costs associated with raising that child because he was negligent and therefore failed to fulfil a general obligation, thus incurring a special obligation to the family. (2) A man lies about his infertility and is coercive, and as a result causes a woman’s pregnancy. The woman is unable to get an abortion since it is illegal where she lives. The man is not an eligible normative father. Therefore, the woman must raise the child herself or give it up for adoption.

While Prusak claims that it does not make sense to talk about compensation for procreation, we commonly think that such examples as these do warrant compensation to normative parents on behalf of children. Compensation for a wrong need not be equivalent to returning someone to their original, ‘unharmed’ state—this is rarely the case when someone is paid compensation—but rather that it is an exchange or substitution for a loss, harm, or damage. I agree with Brake that compensation is not equivalent to normative parenting; child support defrays the costs associated with raising a child and may be owed as compensation when someone has procreated wrongly. Compensation is more intuitively plausible in examples (1) and (2), above. (Even if we do not wish to regard such people as clinicians as ‘procreators’ we can substitute another expression, such as ‘co-creator,’ ‘fabricator,’ or ‘proximate cause of a child’s existence.’) I discuss the obligations of such roles more in the section “Parameters of the amended role responsibility argument.”

I also think that Prusak’s Frankenstein analogy does not show that Victor Frankenstein owed his creation love and other parental obligations. Whilst Frankenstein voluntarily caused the Creature to exist, he did not do so within established social and legal parameters—this shows that he was obligated not to procreate in this way at all. Frankenstein falls into the category of a procreator who is not an eligible parent—he could not ‘parent’ his creation because there are no social or legal norms for *parenting* such a creature. Unlike negligent procreators who owe compensation in the form of child support until the child can function independently in society, the Creature could not be ‘rescued’ (according to a general duty of rescue) even if Frankenstein had wanted to do so; the Creature needed to be part of human society but was unequivocally excluded from it. Against Prusak’s view, I think that the problem with Victor Frankenstein was not that he failed to love his creature or give him normative parenting. Rather, Frankenstein was guilty of violating social and legal norms when he created a life, and in so doing, he failed to consider what his creation’s life would consist of, and whether that life would be decent and worth living.

Therefore, this example does not so much cast doubt on Brake’s understanding of compensation or of a procreative gap as much as it shows that potential procreators have obligations relating to the happiness of their progeny, and this requires that they can be properly socialised, loved, and valued by others. In modern terms, Frankenstein may be equivalent to a doctor in a fertility clinic who creates a human life without ensuring that future person would have a decent life. If a doctor voluntarily created a child with a life not worth living instrumentally for his own ends, such as to satisfy his ambition and vanity, then like Frankenstein, he would be guilty of a greater, more grotesque crime than mere negligence.

In short, to answer the question of who is a normative parent, my response to this agrees with Brake’s. I restate the position as: *A normative parent is someone who voluntarily undertakes the societal and legal parameters associated with parenting, and is socially and legally eligible and capable of doing so, and the child is eligible to be parented by her.* I agree with Prusak, however, that Brake does not substantiate her assessment of compensation due in terms of a procreative gap. I disagree with Brake’s assessment that compensation owed for negligently causing a child to exist should be equivalent to bringing him to a less needy condition until the age of thirteen since this does not adequately differentiate types of procreators and their level of responsibility. In the section “Parameters of the amended role responsibility argument,” I suggest that compensation may be equivalent to outsourcing those parental obligations that permissibly may be outsourced but should be proportionate with the degree to which one is morally responsible for one’s failure to fulfil one’s obligations; not all procreators will owe the same amount, and some will owe nothing.

Applying Brake’s assessment of parenting to the role responsibility argument, a normative parent would not be a morally permissible suicide because she has agreed to fulfil parental obligations herself, and it would be impermissible to contract out these parental obligations to others. Killing herself would prevent her from performing her role-related tasks, as the role responsibility argument suggests. This leaves the problem of how to compensate a child for the loss of a parent who has committed suicide, that I address in the section “Parameters of the amended role responsibility argument.” In the next section, I establish what are essential obligations that preclude permissible suicide on the role responsibility argument.

### The normative content of roles precluding permissible suicide

In this section, I compare and contrast the obligations of normative parents with those of a biological procreator and other roles that also may have normative content to determine what obligations preclude permissible suicide. I argue that some parental obligations may be contracted out and others may not be, and that contractually and voluntarily (either tacitly or explicitly) taking on continuing care and emotional support of someone is what makes suicide impermissible when it would otherwise be permissible. In the section “Parameters of the amended role responsibility argument” I go on to determine what are morally acceptable reasons for someone to be exempted from fulfilling his contractual obligations. These, I argue, are the parameters for morally permissible suicides on the role responsibility argument.

### Obligations of normative parental roles

Hardimon’s and Brake’s views suggest that normative parents have contracts with society. Roles such as parenting are an exchange between social institutions and individual people within those institutions—parents benefit from accepting the institutionally established role, and the institution (society) benefits from someone filling that role and providing the functions associated with it. The details of the specific tasks involved in being a normative parent may vary according to social and legal norms. However, we may be able to distinguish at least some parental obligations that are general. I agree with Brake that voluntary acceptance of parental obligations is necessary, and “that the child be eligible to be parented.” (Brake 2010, p. 3) For instance, one is not a normative parent of a child that one has stolen from her social and legal parents; in that case, one would have an obligation to return the child to her normative parents.

I agree with at least some of the other general parental obligations that Brake proposes:

Our society and legal system assign parents a long support period with responsibilities for more than ensuring mere survival to independence and a minimally decent life—more, that is, than procreative costs. Bringing a child to self-sufficiency—repairing their neediness – does not include the warmth and affection until late adolescence which parents morally owe to children… [In contemporary Western society and legal systems] not only are parents expected to provide eighteen years of support, warmth, and affection, they are expected to enrich children’s lives and seek to enable them to flourish. (Brake 2010, p. 9)

Brake also claims that normative parents may not outsource the tasks associated with bringing a child to independence in a minimally decent life. She says, “parents owe their children a rich, intimate, daily personal relationship; unlike a contractor who oversees (but does not himself carry out) labour, parents must perform a large part of the work of parenting themselves.” (Brake 2010, p. 10) It seems that the crux of Brake’s objection to outsourcing, as when a parent sends a child off to be cared for by others, is that a normative parent should be closely involved in the daily activities of her child. This seems to assume that this cannot be done if one sends one’s child to a boarding school, for example.

I suggest, amending Brake’s view, that one may outsource a good number of normative parental tasks. The essential point is that there should be a close, continuing relationship between parent and child, and that the parent is intimately involved with overseeing and ensuring the child’s care and happiness. However, there are other tasks that normative parents do not necessarily have to perform themselves but are responsible for ensuring that these things are provided by someone. One may permissibly send a child to school, for example, while continuing to oversee and be responsible for making decisions pertaining to the child’s care. My view is that the tasks that may be outsourced permissibly are what constitute procreative costs, as I discuss subsequently. In addition to the parental obligations that Brake suggests, Prusak adds that parents, including biological procreators, must provide life-affirming experiences; they must reconcile children to their condition and reinforce their value, since they have caused their existence. I maintain that there are ways in biological procreators can ensure this without providing it themselves; for instance, by ensuring that there is a suitable normative parent for the child he helps to create.

A possible concern regarding the view that normative parenting is voluntarily acquired is that if someone takes on the role of a parent raising dependent children but is abusive, he is not parenting in the normative sense. This would imply that he would be a permissible suicide where a good parent would not be, but this does not seem intuitively plausible. My response to this, however, is that if he has dependent children, then either: 1) He has (at least tacitly) voluntarily agreed to parent according to normative parameters. If he has parental obligations to his children that are voluntary and he has no reasonable moral basis to cancel or amend his parenting contract, the fact that he is not parenting within established normative parameters means that he is in violation of his contractual obligations to his children. 2) He is negligent and abusive, though he is nonvoluntarily a (descriptive) role-bearer. If he is nonvoluntarily raising children and is abusive, then he fails in his general duty of rescue and he owes compensation. Either way, he fails to fulfil his obligations further if he then also commits suicide since he leaves dependent children without the goods they need. Therefore, this objection fails to show that this view is implausible. I return to the criteria for impermissibility below, in “Comparing and contrasting parental roles with other normative roles.”

In summary, a child must be eligible (legally and socially) to be parented by the person in the normative parental role. Parental obligations include those tasks that are equivalent to procreative costs and may be outsourced, but a normative parent must perform (and be capable of performing) at least these two things himself and these may not be outsourced to others: 1) Overseeing or being responsible for decisions pertaining to the child’s upbringing and welfare. 2) Providing continuing affection, warmth, a sense of value and of social belonging, such as belonging to a family. The fact that these things cannot be outsourced makes the normative parent irreplaceable to his child, and therefore also normally precludes permissible suicide; I will go on to argue, however, that as we have established moral bases for not fulfilling our contractual obligations under certain conditions, we likewise sometimes have a moral basis for not fulfilling our role-related contractual obligations.

Next, I address the obligations of biological procreators to establish that the role responsibility argument can differentiate obligations of normative parents from people who have procreated but may not provide the same ‘goods’ or normative parenting to their children.

### Obligations of biological procreators who are not normative parents

There are a number of different senses in which one may be a biological procreator; for example, voluntarily, as a gamete donor, fertility doctor, or a contracted carrying mother. I suggest that each of these has its own set of obligations. What someone owes depends upon what he has contracted to perform, and whether he has wronged someone by behaving negligently or contrary to the normative parameters of his role-contract. For example, a doctor in a fertility clinic owes a duty of care relating to developing a viable embryo. A gamete donor may only owe gametes and genetic and medical information related to that donation. A nonvoluntary, negligent procreator, however, may owe financial compensation associated with a child’s upbringing; his obligation is not equivalent to normative parenting, but rather may be equivalent to outsourcing those parental obligations that may be outsourced permissibly.

In most cases, biological procreators must ensure that the child can survive and have at least a minimally decent life. If one is a fertility doctor, and if pre-implantation genetic screening for embryos inheriting a severe degenerative abnormality, then the doctor has obligations regarding such embryos being placed in utero*.* Which genetic abnormalities apply is determined socially and legally, in light of best medical practice, as reflected in the Human Fertilisation & Embryology Authority Code of Practice (2017). A fertility doctor is not required to provide procreative costs if he has performed his obligations to the child within his contractual role. If, however, he is negligent and has not performed within this role, he may owe compensation to the parents on behalf of the child, or to the parents themselves who must raise the child with needs beyond those of children without such disabilities. This reflects why we have ‘wrongful life’ suits when children are born with birth defects due to negligence.

A contracted carrying mother has obligations not to add unnecessary risk to the child’s health. If she does, then she is negligent, which amounts to failing to fulfil a general duty to rescue, as Brake suggests. There may be an obligation not to smoke cigarettes, use harmful drugs, or neglect her own health. Biological procreators may also owe the child an adequate account of his origins, in the form of a medical history and genetic background, for example. It also may require justification; it may matter psychologically to know why one’s procreator was not one’s normative parent, for example. What the story of Frankenstein tells us is that the biological procreator did not have adequate justification for creating a creature whose life could not have intrinsic value. Nor did he give his creation the possibility of life-affirming experiences—he was negligent in seeing to his creation’s well-being adequately.

On my view, whether a biological procreator is a permissible suicide on the role responsibility argument will depend upon whether he owes compensation or other special obligations. For example, even if a biological procreator does owe compensation, he may commit suicide once he provides restitution, though he may owe such things as relevant medical information before doing so. By contrast, normative parents have continuing obligations towards their children that normally preclude permissible suicide, though under exceptional circumstances this may not be the case, as I discuss in the section “Harms, wrongs, losses, and proportionate compensation.”

One could think that a special obligation not to commit suicide may arise because of the particular needs children have that adults do not, or that it is because children have not voluntarily accepted their relationship with their parents. I next show that the role responsibility argument applies not only to parents or procreators as a special class, but is also pertinent to other familial roles involving adults who are mutually obligated to each other not to commit suicide, by virtue of their exchanged promises.

### Comparing and contrasting parental roles with other normative roles

Like normative parental roles, spousal obligations are continuing during the span of the spousal relationship. One can imagine circumstances where marriages are expected to have only a short time; for example, a soldier wounded in a war returns home to die, and he and his betrothed get married even though he is dying. This relationship is, nevertheless, continuing because, as with children, spousal relationships form a shared history with affective ties. Unlike parent–child relationships, spousal obligations exist between mutually consenting adults who each voluntarily acquire their role-related obligations and spouses have mutual, though not necessarily identical, responsibilities towards each other’s welfare. (For the sake of brevity in establishing the differences between the parent–child relationship and spouses, I shall leave aside nonvoluntary spousal roles, such as when people are coerced into marriage other than to stipulate that such marriages would not be voluntary, contractual, just, or morally binding.)

The content of specific spousal obligations will vary according to social norms and the understanding that each person has as the basis of that marriage. Some people voluntarily acquire spousal obligations without formal marriage, such as in civil partnerships. They nonetheless enter into an exchange of promises or agreements or understandings that form the basis of their partnership. The role responsibility argument may determine that suicide is impermissible for spouses, but this will depend upon what the spouses understand their marriage to entail. Even if two people vow to remain married until death, such vows may be ambiguous about suicide. This could mean that suicide is allowed—people can agree to be together until they kill themselves in a suicide pact, for example. If they vow to remain together in sickness and health, this also may be ambiguous about suicide—this may obligate each to care for the other despite illness, but says nothing about whether they are permitted to kill themselves if they become very ill. What then, if anything, precludes suicide in spousal obligations? Spousal relationships are generally obligations of continuing care and emotional support—one cannot fulfil such obligations if one kills oneself. Therefore, according to the role responsibility argument, if one is married then suicide may be impermissible if it constitutes abandonment or failure to honour one’s promise of continuing care and emotional support. This is a feature that spousal obligations share with parental obligations.

Not all roles will preclude permissible suicide; the roles of teacher, doctor, and town official are examples of this. One may still object, then, that as special cases, the roles of parent and spouse are not indicative of any underlying principle to do with contracts, but rather are merely duties that stem from close affective ties. I maintain that this not to be the case since other roles, such as that of a soldier or a nation’s leader, show that the role responsibility argument is not limited to close family relations. A soldier’s contractual role obligates her to follow a commanding officer’s orders, providing those are reasonable (in keeping with a just military code). If that officer orders her not to risk her life, but instead to ensure that a message gets to a field marshal, then the soldier is obligated to do so. If she were to throw herself onto a grenade to save her comrades instead, her suicide would be morally impermissible. If, on the other hand, a commanding officer asks for volunteers for a suicidal mission and a soldier volunteers, then that soldier is obligated in the endeavor of carrying out the mission. If she has no intention of doing so, but is instead planning to desert, this would be impermissible. However, the soldier’s contract is also applicable to intended self-killings; for example, a soldier who disobeys orders and, weary of battle, throws herself into the line of fire to end her life would not be a permissible suicide because she is disobeying orders.

The role-responsibility argument also applies to sublimated or sub-intended self-killings; Captain Ahab in *Moby Dick,* for example, was a sub-intended suicide. (Shneidman 1981, pp. 246–253) As Captain, his role was to ensure the safety of those under his command and fulfil his own contractual duties towards the shareholders of The Pequod, to bring back as much spermaceti oil as possible, as quickly as possible. Ahab also had an obligation not to pursue a suicidal mission whilst in command of his ship. A leader must not order the ship’s helmsman to steer them into rocks or to pursue a suicidal mission that suits his own purposes. If he does so, then he impermissibly fails in his contractual obligations. Ahab committed those under his command to suicide, so his suicidal mission was also homicidal. (Dowie 2020, p. 730)

Leading, coercing, or manipulating people into suicide can constitute a type of homicide. Some roles contain a power dynamic that can be used to manipulate or coerce others into suicide. (Dowie 2020, p. 731) Since, for instance, the role of a soldier obligates him to following his commanding officer’s orders, if that officer wrongfully sends the soldier on a suicidal mission because he covets the soldier’s wife, then the commanding officer has murdered the soldier, as King David murdered Uriah. Such examples involve a violation of one’s contractual role as a leader, since morally binding contracts require fiduciary obligations; these leaders do not operate within, but rather contrary to, normative parameters of the role of leader. A government has continuing duties of care and support towards its people, and the leader of a government, by extension, operates according to this principle.

In summary, I suggest amending the role responsibility argument to: P1) If someone has a role-related obligation to provide specific people with continuing care and support, then they have strong moral reasons not to cease providing this role-related care and support. P2) Normally, suicide causes a situation in which people cease to provide care and support. Conclusion: *If someone has a role-related obligation to provide specific people with continuing care and support, then normally there are strong moral reasons against that person committing suicide.* This understands roles as having normative parameters that consist of contractual, (at least tacitly) voluntarily acquired special obligations. If one has nonvoluntarily-acquired obligations, such as those of an accidental procreator, or if one has discrete role-related obligations, then suicide may be permissible when one discharges these duties.

There may be morally acceptable reasons not to fulfil one’s special role-related obligation not to commit suicide, even if that obligation is voluntarily acquired. I next address under what conditions a suicide would be permissible even though one’s role normally precludes suicide, and those conditions under which suicide remains morally impermissible and/or blameworthy; I clarify what constitutes normal conditions that preclude permissible suicide and exceptional ones that permit it.

## Parameters of the amended role responsibility argument

I propose that as we have established morally acceptable criteria for cancelling, voiding, or amending a contract, we can apply these to the role responsibility argument to establish grounds for permissibly not fulfilling one’s role-related obligations. A parent who is terminally ill, for example, or has other morally compelling reasons for failing to fulfil her role-related obligations may permissibly kill herself. In this section, I suggest that the same criteria that determine culpability in homicide and suicide also determine culpability for failing to fulfil one’s role-related obligations; these include intention, voluntariness, diminished responsibility, mental capacity, and foreseeability. (Dowie 2020, 2022).

### Criteria for amending, cancelling, and voiding one’s role-contract

We have general duties not to cause harm, injury, or loss. We acquire additional special obligations voluntarily through promises and contracts that generate reasonable expectations in others that they then act upon. When promises and contracts are broken, people can be wronged consequently; for example, if someone enters into a contract to build ten pieces of equipment, she must lay out capital to buy the components to build it. If the other party then cancels the contract, the builder may suffer financial loss. Even if the promisee is not made worse off, and even if the promissor never intends to honour it, it seems that the promissor ought to honour that promise. In *Promises, Morals, and Law,* P.S. Atiyah points out, “A man may *think* that he ought to fulfil a promise, but his thinking that he has an obligation cannot *be* the (sole) source of the obligation; if so, only honest men would be bound by promises.” (Atiyah 1981, p. 18) Promises, he suggests, must be grounded in something external from mere thoughts or intentions.

Atiyah suggests that while we have internal, intuitive reasons for honouring them, we also have socially derived reasons for enforcing promises and contracts. He notes that mischiefs arrive from not honouring or enforcing them. One way to protect people from wrongs is through juridical law. However, he also acknowledges that not all promises or contracts are binding, such as if the promissor lacks mental capacity, is a child, is involved in fraud, coercion, or illegal or immoral acts, or where there exists a ‘deficiency.’ (Atiyah 1981, p. 22) Deficiency may amount to overwhelming coercive pressure; as an example of this, he discusses a man on a vessel throwing goods overboard in a storm. Although the man may have had every intention of getting the goods safely to dock, circumstances, such as an unexpected storm, may prevent him. Atiyah explains that promises are conditional, then, on a set of circumstances being in place. (Atiyah 1981, p. 22).

For one thing, if ‘ought implies can,’ then promising the impossible is not a binding promise. (Atiyah 1981, p. 26) This means that not all promises are binding, but may be binding by degree, since circumstances are in place by degree. It is not clear that keeping a promise ought to be privileged over the promissor’s right to change his mind, or that promises are any more binding than other sorts of stated intentions. Threats, for example, can be seen as stated intentions, but do not seem to be morally binding. Ross and Stratton-Lake argue that while we have a *prima facie* duty (he thinks that this is nonetheless a real duty, contingent upon circumstance) to keep promises, such duties sometimes conflict with other duties, such as to help others. What we ought to do amounts to a “compromise between the true notion of the right act and the notion of the morally good action.” (Ross and Stratton-Lake 2002, p. 32) He argues that our duty, then, may be to keep promises but so long as we do not have an over-riding reason not to, such as because the consequences of doing that duty harms someone else badly.

I propose that, as with other contracts, role-related contractual obligations, including those of parents, have a moral basis to be modified, depending upon the circumstances. I stipulate that while we may have a duty to keep promises, not all failures to keep promises constitute a wrong or warrant restitution; someone could promise to take his daughter to the park, but seeing that she is happier going with a friend instead he suggests that they go without him—he has not wronged her. Enforcing a promise or contract may place an undue burden on a promissor, and this may be unjust if the promisee has not been harmed and/or the promissor is not at fault for his failure. Because of such concerns, the conceptual framework of contracts applies the principles of proportionality and reasonableness to balance the conflicting interests of contracting parties. I appeal to the conceptual framework of contracts to provide guidance about more difficult cases of suicide with respect to the role responsibility argument; the principle of proportionality balances the limits of promissory obligations against reasonable expectations, as discussed further in “Harms, wrongs, losses, and proportionate compensation” (below).

There are several morally acceptable reasons to modify an existing contract in the conceptual framework of contracts, including the following, though these may not be exhaustive: (1) If the parties involved agree to terminate or alter the role-related obligations and they have the mental capacity to make this decision; (2) If the laws (or social norms) change, then the contract would be replaced by a new one; (3) If the contract is unjust, then the contract could be voided; (4) If someone lacks capacity when he enters into a contract, or if it was entered into under duress, then that contract is voidable or liable to be cancelled or amended; (5) If circumstances have changed and fulfilling the contract is too great a burden, according to the principles of proportionality and reasonableness, on the contracted agent to carry out, such as if he is sufficiently ill, then the contract can be altered or terminated. (For outlines of defensible breaches, see The Juridical Education Center at The University of New Mexico’s “Defenses to Breach of Contract,” (Juridical Education Center 2018) and the State of California’s court document “Affirmative Defenses—Contract” outlining contract. (California State Courts 2011)) I explain these more below, in connection with the role responsibility argument:

1. People may renegotiate or cancel contracts if the parties involved voluntarily consent to do so. This means that if someone has a role as a spouse and wishes to commit suicide and her spouse agrees, then it may be permissible not to fulfil one’s role-related obligation not to commit suicide. One possible objection may be that the husband of a suicidal agent, for example, ought to prevent her suicide—it could be callous to permit it. Worse, he may wish her dead and use this as a convenient means of ridding himself of her. Intuitively, we may think that people are obligated to prevent a suicidal spouse’s death. If we consider, however, that morally binding contracts contain fiduciary obligations, and that there may be morally compelling reasons for a husband to voluntarily relieve his wife from her role-related obligations, this objection may not carry as much weight as it appears to on the surface. For example, one’s wife may be suffering from a severe, long-term illness that devalues her life, and she has a settled intention to die. A husband may excuse her from spousal obligations out of beneficence.
2. There are also circumstances where the parties do not need to agree to have contracts voided or altered. Contracts may become void or amended due to changes in law. For parents, this means that if societal parameters for acceptable parenting change, or the laws change, then their contracts change. If, for instance, Scotland had adopted the controversial ‘Named Person Scheme,’ parents would be required to allow a social worker, for instance, to monitor their child, thereby changing the parental contract. (Nicolson 2017) In terms of suicide, part of a doctor’s contractual obligations includes not assisting a patient in suicide. If the laws and professional codes were to change such that they were to allow this under certain conditions, then the doctor’s prior contractual agreement would no longer be binding but would be replaced by the new contract/professional and legal code pertaining to physicians’ duties.
3. Contracts that do not include fiduciary obligations are unjust, not considered binding, and may be voided. Hardimon’s position that people have an obligation to carry out the tasks associated with their role, providing that the institution of which the role is part is just, incorporates fiduciary obligations into his conception of contractual role obligations. This means that one may permissibly void one’s role-contract if it is unjust, intrinsically unfair, wrong (it is based upon doing something malevolent), or it creates an imbalance. For instance, a contract whereby one person enslaves another is not just, and is void.

If a contract is based upon a mistake or misrepresentation, then one or both parties may void the contract without being morally culpable for breach of contract. (Hillman 1982, p. 690; Meier 2017, p. 14) For instance, as in a recent court case, partners agreed to have a child together with the aid of IVF, but then split up some time later and the boyfriend changed his mind about the procedure. The girlfriend forged her ex-partner’s signature on the paperwork for the fertility clinic and misrepresented his current wishes to the clinic. (The Telegraph 2017) This biological father would not be responsible to compensate the mother on behalf of the child since this act of parenting a child was based on fraud. Other acts of procreation may be morally similar, such as if someone lies about contraceptives or infertility and pregnancy ensues.

(4) Contracts are also voidable or permissibly cancelled if they are made under duress or undue influence or if one of the parties lacks capacity to enter into it. (Meier 2017, p. 14) This means that contracts are not binding on children since they lack capacity to make these sorts of decisions. It also means that if someone is in a psychotic episode when he enters into a contract, it may be cancelled or voided. A woman who is institutionalised for mental disorders may not be an eligible parent because she lacks capacity to carry out the role-related duties; her child may be given to normative parents to raise, at least temporarily. If someone has full mental capacity to enter a contract but subsequently loses her capacity, then this may be a moral justification for amending or cancelling it, depending on the degree of loss involved, if she still has at least some capacity for making certain types of decisions and performing actions pertaining to her role, and the nature of that contract. (There may be exceptions to this, however, as with an advance decision refusing life-saving treatment; see Dowie 2019). As already noted, impermissibility does not imply blameworthiness; one may have diminished responsibility that mitigates blame for failure to honour one’s contractual obligations.
(5) There may be cases where someone is in breach of his role-related obligations, but binding him to fulfilling those obligations is unjust because his circumstances have changed significantly. In contract theory, this is called impossibility, impracticability, or frustration, depending upon the circumstances in the case. This also may be due to a mistake, such as if someone enters a contract without adequate knowledge of the facts at hand. (Hillman 1982, p. 690) For example, a father with dependent children develops a brain tumour. He suffers from pain, personality change, and periods of erratic behaviour. He nevertheless has contractual obligations to his children, but it would not be reasonable to expect him to be able to fulfil those obligations as if he was not ill. This is for two reasons: (1) His physical and mental ability is impaired, even though it is not lost. (2) He has diminished responsibility in performing his contractual obligations and doing so would be an unreasonable burden. Nevertheless, he may be a permissible suicide since he has mental capacity to decide this (or has established an advance decision refusing life-saving treatment; see Dowie 2019), and his contractual role-related obligations have adequate grounds for amendment.

Even when there are moral grounds to amend a role-contract, people still may have obligations to make alternative arrangements for others, for example. Failure to fulfil one’s contractual role-related obligations where one does not have an adequate basis would remain morally impermissible. This may not be blameworthy, however, depending upon the circumstances. Next, I discuss when people may be culpable for their failure to fulfil their role-related obligations for providing continuing care.

### Failure to fulfil role-related duties—causation and culpability

A person can acquire obligations to someone else by being a proximate cause of a harm, as discussed previously. (This may be determined according to the *sine qua non* rule in legal philosophy; it would not have happened but for the act or omission of an agent.) Alternatively, she acquires obligations to someone, such as a spouse or a child, because she has voluntarily entered into a role-contract. This means that she has promised to carry out role-related responsibilities. Since promises generate expectations, and are relied upon, we have reasons to enforce them. Since, however, misunderstandings can happen, despite best intentions, and we cannot always foresee circumstances that make honouring those promises impossible, we rely on the principle of reasonableness. This is, roughly, how would an impartial, reasonable person interpret the terms of a contract, or what would a reasonable person in similar circumstances reasonably expect.

This is similar to William Frankena’s position that reasonable people taking a moral point of view would arrive at a consensus, given all of the relevant facts. (Frankena 1973) If we apply the principle of reasonableness to the role responsibility argument, this would tell us when a parent is failing to fulfil her role-related contract, or is negligent in her duty of care to her child, by appealing to what other reasonable parents would do under similar circumstances. A parent has violated her role-contract if other reasonable normative parents, under similar circumstances, would have acted differently or would have interpreted the parameters of the role differently. If they would have done the same, then the parent did not violate her role-contract. If she has violated her contract, there are degrees of culpability, similar to other failures to carry out one’s obligations.

Whether one has actively done something or passively allowed it to happen is sometimes but not always relevant to culpability; passively neglecting one’s children may be as bad as actively abusing them, but that may depend upon other factors, such as the extent of the abuse/neglect. To be culpable for such things as sublimated or passive suicides that violate one’s role-related obligations, the death must be reasonably foreseeable and constitute negligence; for example, a parent who fails, through negligence, to receive life-saving treatment for an easily treatable infection and dies as a result may be culpable for violating her role-related contractual duties to her children. Reasonable parents would not neglect their health in this way, with their deaths as a reasonably foreseeable consequence and without adequate regard for their children’s welfare; hence, this mother violates her role-contract through neglect.

A community that raise children communally, as Brake discusses, may have corporate role-related contractual obligations. If this community were comprised of suicidal cult members, like the parents at Jonestown, by killing themselves they would constitute a collective suicide that violates their role-related obligations to their children and there would be multiple responsible agents. Depending on the circumstances, a ringleader who instigates this sort of suicide or mass suicide may be deemed to be more culpable than the others in the gang who also cause the deaths. For example, at the Jonestown Massacre, Jim Jones was the principal agent who instigated mass suicide and mass homicide. His followers acted as a collective of accomplices who violated their role-related obligations to their children. All of these people at Jonestown also had a general obligation to these children not to lead them to their deaths and were obligated not to follow Jones, though some of these people may be more culpable than others. (Compare this to a corporation who has violated its contractual or moral obligations; some of the principals may be more culpable than others, depending upon the authority and circumstances.)

I next discuss how the criteria of intention, voluntariness, diminished responsibility, mental capacity, and foreseeability are relevant for establishing the level of culpability for breach of contracts and contractual role-related obligations.

### Failure to fulfil role-related duties—intention and foreseeability

Intention impacts the moral characterisation of an act, and this includes an act of suicide. (Dowie 2020, 2022) Intention should also be considered as morally relevant in a failure to fulfil role responsibilities. Someone could act according to mistaken beliefs when he fails to fulfil his contractual duties—perhaps he mistakenly believes that his actions will not wrong anyone. If, for instance, a father who is suffering from schizophrenia kills himself, wrongly believing that his wife and children are better off without him and would prefer that he died, his intention is not to abandon his family. A father also could kill himself because he mistakenly believes that his family has been killed in a flood; again, his intention is not to abandon them.

A parent can have good intentions in breaching her parental contract, and other duties may outweigh her contractual parental duties. For example, the World War II hero Violette Szabo despaired at the loss of her husband, so she directed her grief by volunteering for dangerous duty as a secret service agent. Szabo knew that it was foreseeable, even highly likely, that in doing so she would orphan her child, whose father had already been killed in the war, but she left her daughter with relatives to raise. Szabo was then tortured and killed by the Germans. She was not negligent to her daughter, as she had arranged for others to care for her. She also acted within the framework of the rules of warfare. While her motives seem to suggest that she acted out of grief, the Crown had reasons pertaining to national morale during wartime to promote this sort of action as exemplary and self-sacrificial; the juridical and social framework during the war suggested that getting oneself killed was not construed as abandonment but rather as self-sacrifice. Szabo was awarded the George Cross posthumously; King George VI presented her daughter with it. Szabo did, however, intentionally, voluntarily, and permissibly ‘outsource’ her voluntarily-acquired child-rearing obligations. One could also argue that she fulfilled normative parental obligations of continuing care and emotional support, even after her death, in light of her legacy.

As with ascribing responsibility and culpability generally, the matter of whether one acts voluntarily or nonvoluntarily and whether one is coerced may impact the degree to which someone is culpable when she violates contractual role responsibilities. Many of Jim Jones’s followers, for example, would probably amount to either voluntary or involuntary manslaughter as well as self-manslaughter, since many of the adults were vulnerable, brainwashed, or at least manipulated; some would be more culpable than others. Some of these parents even may have been morally equivalent to a mother who drives herself and her children off a cliff into the sea because she suffers from severe post-partum depression—while doing so constitutes wrongful homicide as well as suicide, she may not be entirely culpable. (Dowie 2020, 2022)  These may be cases of voluntarily failing to fulfil one’s role-contract, but with diminished responsibility, as I discuss more below in the next section.

Foreseeability is relevant to whether one should be held morally responsible and/or culpable for one’s failure to fulfil one’s role-related obligations. If it is reasonably foreseeable that acting (or omitting to act) violates one’s contractual obligations then this would not be permissible, as with the mother who fails to treat her easily treatable infection and dies as a result, leaving her children uncared for. Similarly, if someone accidentally but foreseeably kills himself while driving under the influence, then he is negligent to his children. The driver has no moral grounds to void a parenting contract he made in relation to his children—his self-killing due to driving drunk is a reasonably foreseeable consequence of a voluntary act of getting drunk and then driving. He would wrong his children if he killed himself in this manner and would be culpable. Unlike the mother who fails to treat her infection, the drunk driver also wrongs society because in acting this way he endangers others and is potentially homicidal as well as behaving suicidally. (Dowie 2020).

### Accident, mistake, and negligence in failure to fulfil role obligations

One may fail to fulfil one’s role-related obligations due to accident or mistake. As Brake suggests, for example, one may cause a child’s existence accidentally or negligently. Accidental procreation, such as when one uses contraception that fails and pregnancy ensues, may not be culpable, but negligent procreation, where someone is apathetic about using contraception, would be culpable. I suggest that as we distinguish between unintended deaths that are negligent and deaths that are accidental or mistaken (Dowie 2020), we can do the same for failure to fulfil contracts or other obligations. Even if an act is not culpable, failure to fulfil obligations due to accident or mistake may owe compensation, as I address in the next section, but may owe less than acts due to negligence.

### Diminished responsibility in failure to fulfil role obligations

Failure to fulfil one’s contractual role-related obligations where one does not have morally acceptable reasons for amending one’s role-contract may be morally impermissible. Impermissibility does not necessarily imply blameworthiness, however. One may be culpable by degree. Whether we hold someone morally responsible for fulfilling his contractual obligations may depend upon whether he had the capacity to make such decisions voluntarily, if he acted with diminished mental capacity or diminished responsibility, or there may be other extenuating circumstances, such as if he has a severe illness. For example, while a woman who has post-partum depression may constitute a morally impermissible suicide, her death may not be blameworthy because she has diminished responsibility.

When we consider the reasons that many people commit suicide, such as believing that their options are very limited or non-existent, due to mental impairment, or because they have been coerced or manipulated by others, we may think that many suicides are performed with diminished responsibility, making the deaths involuntary self-manslaughters or voluntary self-manslaughters, rather than self-murders. (Dowie 2020) Even so, this leaves the problem of how to compensate a child, for example, for the loss of a parent who has committed suicide. Next, I propose an amendment to Brake’s assessment of compensation.

### Harms, wrongs, losses, and proportionate compensation

In the conceptual framework of contract theory, the principles of proportionality and reasonableness protect people who are incapable of fulfilling their contractual obligations, and those to whom they are obligated, by holding people only to fulfilling what can be reasonably expected under the circumstances. Actual harms resulting from one’s failure to fulfil a contract must be assessed. Compensation, or restitution, also must be assessed. The principle of proportionality sets limits to restitution for failing to fulfil one’s obligations. The principle of reasonableness relates to how a reasonable person would interpret the terms of a contract, or what would he expect in similar circumstances. These principles balance conflicting interests between the contracting parties. (Faccio 2014, p. 200) The amount of compensation owed depends upon the degree of wrong-doing caused, whether one has done so voluntarily, what one’s intentions were, whether one suffers from diminished responsibility or mental impairment, and whether the wrong was a reasonably foreseeable consequence of one’s acts and/or omissions.

Applying these principles to the role responsibility argument can balance the conflicting needs of parents in defensible breach of contract with those of their children, for example. For children who have lost a parent, the harms done will be great, and include emotional well-being as well as other welfare considerations. The children, rather than society, would be the party wronged in such a failure to fulfil one’s parental contract, but children cannot voluntarily enter into or negotiate contracts. Therefore, society would act on their behalf in terms of acting on their behalf; children would be third-party beneficiaries. Compensation may include determining who will care for the child as a normative parent.

If the terms of one’s role-related contract are fair, acquired voluntarily with full mental capacity, and there are no moral grounds to void, cancel, or amend the role-contract, then failure to uphold the contract constitutes a moral impermissibility. For example, O.J. Simpson had a role-related obligation to his children to ensure that they received normative parenting. In the civil case brought against him for murdering his wife Nicole, the children and Nicole’s family were awarded many millions of dollars in compensation for their loss. If Simpson had not been wealthy, or if the circumstances had mitigated his culpability, then the principle of proportionality would have awarded the family less. The war hero Violette Szabo, by contrast, did not owe compensation to her daughter even though she acted knowing that it was likely that she would orphan her; Szabo did not abandon or wrong her daughter, she made adequate provision for her, and her actions were socially and juridically sanctioned, and were morally justifiable under the circumstances.

Whilst it would be nonsensical to think that compensation brings a child to its original ‘unharmed state,’ it is aimed at making reasonable reparations and mitigating damages. This raises the question of what happens to children if their parents do not or cannot provide alternative normative parenting and/or compensation. If there are no voluntary normative parents for such children, then the state seeks suitable normative parents, asking relatives to volunteer or assigning children to foster-care. Additionally, in the United States, for example, if a minor loses a parent, then the Social Security Administration pays the child and/or the remaining (or replacement) normative parent on behalf of the child. This is intended to compensate for the loss by assisting with costs associated with outsourcing those tasks that can be outsourced—providing clothing, medical care, and education.

Society, then, has a general duty of rescue to children who are harmed and/or wronged by their parents’ deaths, whether this death is a suicide or not. This is intuitively just if we consider that society has a contract with parents and as such also has promissory obligations. The way in which society carries out this general duty of rescue will depend upon the roles of its members—as Brake suggests, society determines child-care responsibilities, including assigning eligible normative parents. Returning to Prusak’s objection to Brake’s position, I think that society owed a general duty of ‘rescue’ for the wrongs that Victor Frankenstein inflicted on the Creature who had no choice but to be created wrongfully and mistreated. In this case, we may even think that the Creature was owed compensation not just from Victor Frankenstein, but also from society—the Creature was alienated and marginalised in a way that made his plight even worse.

I suggest that society’s general obligation of rescue to children includes: a) To ensure that standards of the role of parenthood are adequate to providing a decent standard of living for children generally. This means that it ensures appropriate levels of well-being, education, and healthcare needed to bring a child to full independence. b) To enforce that parents operate within the normative standards of the role of parent. c) To establish and enforce compensation for failures of fulfilling normative parental obligations. If a nonvoluntary procreator from an economically disadvantaged background cannot provide full compensation for negligent procreation, society may have an obligation to provide the difference for social reasons.

My proposal says that a parent may permissibly commit suicide sometimes, leaving behind her grieving children, based on contractual considerations. I agree with Cholbi when he claims “to have a loved one die by suicide is often a painful experience that can probably not be adequately captured in terms of the abstract talk about duties, rights, and so forth.” (Cholbi 2011, p. 62) However, my proposed amendment to the role responsibility argument is much like those made in the context of divorce, adoption, probate, in claims of damages, etc*.* While judgements of duties and rights do not express the experiences of those involved, they do give us a practical basis for establishing what ought to be done and why. We have social reasons to enforce obligations towards children; society ought not to allow vulnerable people be abandoned by their care-takers. Children’s interests must be balanced with the needs of parents who are also vulnerable because they are suffering. Society, however, has a responsibility to remedy the deficiency of parents. Additionally, we may stipulate that a general obligation of rescuing a child whose parent has committed suicide is addressing special needs that they have as a result, such as assistance with emotional distress, etc*.* that arises with such a profound loss.

One may object to my proposal on the grounds that moral and juridical laws are not equivalent, and that juridical laws do not inform us of moral ones. However, I do not base this on juridical law, but on the moral and conceptual framework underpinning contract theory within legal philosophy. Furthermore, the conceptual framework offered here is based upon moral considerations of reasonableness, beneficence (in terms of fiduciary obligations) and justice, as Hardimon points out. Therefore, I do not think that this objection shows my proposal to lack moral foundation.

### Nonvoluntary roles: a possible objection and my response

A possible objection to my position is that someone could think that it does not allow for nonvoluntary, non-contractual normative roles that we are born into, such as being a sibling, a child, or a citizen. Hardimon says that non-contractual roles are more pervasive than people generally believe. He thinks that children and citizens fall into this category since they do not voluntarily choose to be born into a particular institution (including families as social institutions). (Hardimon 1994, p. 342) If one takes up Hardimon’s view that at least some normative roles are nonvoluntary and non-contractual, such as being a child or a citizen, then it would not make sense to regard such roles as having the contractual form of offer, acceptance, and exchange of promises; these roles are obligatory based entirely on such things as one’s being born at a particular place and time, and to particular people. If these are not voluntarily entered into or contractual, we may not have a basis for explaining how and why they entail special obligations.

However, whilst I do not deny the possibility that some non-contractual nonvoluntarily-acquired normative roles exist, they do not show that my position is implausible. Brake suggests that what seem to be non-contractual normative roles, such as children having duties to parents, could be explained by virtues, rather than in accordance with nonvoluntarily-acquired obligations that children have towards parents due to their role as children. I also suggest, as an alternative to Brake’s view of virtue, that roles such as those of children and citizens could be regarded as descriptive, not normative roles. We can say that adult offspring have contractual obligations to their parents only when they develop the capacity to enter into such a contract voluntarily. Until then, a child’s role is not normative but rather is descriptive. In other words, their role in relation to their parents changes when they become independent and they only enter into contractual obligations to care for their parents voluntarily.

Hardimon points out that institutions must be just and hypothetically acceptable. I suggest that nonvoluntary roles must be assented to in order for them to be just. This means that in an institution like a country, a citizen, while not consenting to her birthplace, must be a willing and tacitly voluntary participant, or we risk injustice to citizens. The special obligations that citizens have towards their country or countrymen may be explained, similar to nonvoluntary procreators, as arising from general duties. A citizen may acquire special obligations, such as that of compensation or a ‘debt to society,’ if she does not fulfil a general duty to obey the law.

However, even if some normative roles are nonvoluntary and non-contractual, it is not clear that the role of a citizen, for example, would preclude permissible suicide according to the role responsibility argument as the normative role of parent would—compensation is a discrete obligation and it would seem that one may permissibly kill oneself once one discharges one’s duty. A claim that we have nonvoluntarily acquired obligations to society not to commit suicide because it deprives society of the goods that one produces is questionable at best; people can be a drain on society rather than a benefit. (Cholbi 2011, p. 60) Furthermore, we have good reasons to believe that maximizing overall well-being levels can be unjust. (See Broome 1984 for a critique of consequentialism and the Rawlsian Difference Principle.)

## Conclusion

I have clarified that the role responsibility argument against suicide’s permissibility should be amended as follows: *If someone has a role-related obligation to provide specific people with continuing care and support, then normally there are strong moral reasons against that person committing suicide. *This understands roles as having normative parameters that consist of contractual, (at least tacitly) voluntarily acquired special obligations. If one has nonvoluntarily-acquired obligations, such as that of an accidental procreator, or if one has discrete role-related obligations, then suicide may be permissible if and when one discharges these duties. I have suggested that the conceptual framework of contracts provides guidance about more difficult cases of suicide with respect to the role responsibility argument. I proposed several morally acceptable reasons to modify an existing contract, including: (1) If the parties involved agree to terminate or alter the role-related obligations and they have the mental capacity to make this decision; (2) If the laws (or social norms) change, then the contract would be replaced by a new one; (3) If the contract is unjust, then the contract would be void; (4) If someone lacks capacity when he enters into a contract, or if it was entered into under duress, then that contract is voidable or liable to be cancelled or amended; (5) If circumstances have changed and fulfilling the contract is too great a burden, such as if he is sufficiently ill. The principles of proportion and reasonableness balance the conflicting interests of duties to others and one’s own liberties. Even when suicide is not permissible it is often not blameworthy given the extenuating circumstances underlying an agent’s suicide, and when we consider that the contractual obligations we have not to kill ourselves are provisional.
